# Donepezil suppresses intracellular Ca^2+^ mobilization through the PI3K pathway in rodent microglia

**DOI:** 10.1186/s12974-017-1033-0

**Published:** 2017-12-22

**Authors:** Yoshinori Haraguchi, Yoshito Mizoguchi, Masahiro Ohgidani, Yoshiomi Imamura, Toru Murakawa-Hirachi, Hiromi Nabeta, Hiroshi Tateishi, Takahiro A. Kato, Akira Monji

**Affiliations:** 1 Department of Psychiatry, Faculty of Medicine, Saga University, 5-1-1 Nabeshima, Saga, 849-8501 Japan; 2 Department of Neuropsychiatry, Graduate School of Medical Sciences, Kyushu University, 3-1-1 Maidashi, Higashi-ku, Fukuoka, 812-8582 Japan

**Keywords:** Microglia, Calcium, Donepezil, Alzheimer’s disease, Phagocytosis, Nitric oxide

## Abstract

**Background:**

Microglia are resident innate immune cells which release many factors including proinflammatory cytokines or nitric oxide (NO) when they are activated in response to immunological stimuli. Pathophysiology of Alzheimer’s disease (AD) is related to the inflammatory responses mediated by microglia. Intracellular Ca^2+^ signaling is important for microglial functions such as release of NO and cytokines. In addition, alteration of intracellular Ca^2+^ signaling underlies the pathophysiology of AD, while it remains unclear how donepezil, an acetylcholinesterase inhibitor, affects intracellular Ca^2+^ mobilization in microglial cells.

**Methods:**

We examined whether pretreatment with donepezil affects the intracellular Ca^2+^ mobilization using fura-2 imaging and tested the effects of donepezil on phagocytic activity by phagocytosis assay in rodent microglial cells.

**Results:**

In this study, we observed that pretreatment with donepezil suppressed the TNFα-induced sustained intracellular Ca^2+^ elevation in both rat HAPI and mouse primary microglial cells. On the other hand, pretreatment with donepezil did not suppress the mRNA expression of both TNFR1 and TNFR2 in rodent microglia we used. Pretreatment with acetylcholine but not donepezil suppressed the TNFα-induced intracellular Ca^2+^ elevation through the nicotinic α7 receptors. In addition, sigma 1 receptors were not involved in the donepezil-induced suppression of the TNFα-mediated intracellular Ca^2+^ elevation. Pretreatment with donepezil suppressed the TNFα-induced intracellular Ca^2+^ elevation through the PI3K pathway in rodent microglial cells. Using DAF-2 imaging, we also found that pretreatment with donepezil suppressed the production of NO induced by TNFα treatment and the PI3K pathway could be important for the donepezil-induced suppression of NO production in rodent microglial cells. Finally, phagocytosis assay showed that pretreatment with donepezil promoted phagocytic activity of rodent microglial cells through the PI3K but not MAPK/ERK pathway.

**Conclusions:**

These suggest that donepezil could directly modulate the microglial function through the PI3K pathway in the rodent brain, which might be important to understand the effect of donepezil in the brain.

**Electronic supplementary material:**

The online version of this article (10.1186/s12974-017-1033-0) contains supplementary material, which is available to authorized users.

## Background

Microglia are immune cells which are derived from progenitors that have migrated from the periphery and are from mesodermal/mesenchymal origin [1]. After invading the brain parenchyma, microglia transform into the “resting” ramified phenotype and are distributed in the whole brain. However, microglia revert to an ameboid appearance when they are activated in the disturbances including infection, trauma, ischemia, neurodegenerative diseases or any loss of brain homeostasis [2, 3]. Microglia are the most active cytokine-producing cells in the brain and can release many factors including pro-inflammatory cytokines (such as TNFα, IL-6), nitric oxide (NO), and neurotrophic factors (such as BDNF) when they are activated in response to immunological stimuli [4–7].

Alzheimer’s disease (AD) is the main cause of dementia and affects 46.8 million people worldwide [8]. According to the “amyloid cascade hypothesis”, abnormal deposition of amyloid-β (Aβ) peptides in the brain cause plaque and tangle formation, neuronal and vascular damage, cell loss, and finally dementia [9]. In contrast, increasing evidence suggests that the pathogenesis of AD is not restricted to the neuronal compartment, but is strongly interacted with neuroinflammation induced by senile plaques and/or neurofibrillar tangles [10]. In AD, Aβ peptides can directly stimulate microglia to release TNFα and NO [11] and the activation of microglia is supposed to promote neuroinflammation resulting in the neurodegeneration [12]. Recently, acute stress is shown to increase the amount of TNFα produced by ramified microglia in the hippocampus of rodent brain. In addition, administration of etanercept, a TNFα inhibitor, can prevent the deficits of spatial working memory induced by acute stress in accordance with a decrease in the expression of TNFα in rodent hippocampus [13]. Thus, TNF-α inhibitors might slow down cognitive decline and improve daily activities of patients suffered from AD through the modulation of microglial functions [14].

As one of underlying mechanisms of aging and AD, dysregulation of intracellular Ca^2+^ homeostasis has also been proposed as a common cause of neural dysfunction [15, 16]. However, some reports suggest that dysregulation of intracellular Ca^2+^ is not restricted to neurons but represents a global phenomenon affecting glia including microglial cells in the brain of AD [17]. Donepezil, one of acetylcholinesterase (AChE) inhibitors, is clinically used for the treatment of AD [18]. The major mechanism of donepezil’s effects is to inhibit AChE activity thereby increasing the acetylcholine (ACh) levels. ACh then promotes the neuroprotection against glutamate-induced excitotoxicity by stimulating the phosphatidylinositol-3 kinase (PI3K)/Akt pathway [19]. Notably, donepezil has also shown to have anti-inflammatory effects in the brain of experimental animal models. In amyloid precursor protein (APP)/presenilin-1 (PS1) transgenic mice, treatment with donepezil significantly improved the cognitive function and suppressed the expression of CD68, a specific marker of microglial activation [20]. Pretreatment with donepezil significantly suppressed the production of TNFα and NO induced by LPS [21] or by Aβ peptides [22] in rodent microglial cells. In addition, Hwang et al. showed that donepezil-induced suppression of microglial activation does not depend on α7nAChRs [21].

To our knowledge, this is the first report to examine whether pretreatment with donepezil affects the intracellular Ca^2+^ mobilization and to test the effects of donepezil on phagocytic activity in rodent microglial cells.

## Methods

### Materials

The drugs used in the present study include Fura2-AM, adenosine 5′ triphosphate (ATP), donepezil, acetylcholine, methyllycaconitine (MLA), BD1047, BD1063, LY294002, U73122, PD98059, KN-62, 4,5-diaminofluorescein diacetate (DAF-2DA), and human recombinant TNFα were purchased from Sigma-Aldrich (St. Louis, MO). MSPG [(R,S)-α-2-methyl-4sulfonophenylglycine] was purchased from Tocris Bioscience (Bristol, UK). Human recombinant TNFα was diluted with the standard external solution to obtain the final concentration. Donepezil was diluted with the standard external solution to obtain the final concentration (5 μM). This donepezil concentration is sufficient to inhibit the AChE activity in both human blood cells and monkey brain samples [23] or to suppress the LPS-induced NO production in mouse primary microglial cells [21]. Drugs that were insoluble in water were first dissolved in dimethylsulfoxide (DMSO; Wako Pure Chemical Industries, Osaka, Japan) and then diluted in the standard external solution. The final concentration of DMSO was always less than 0.1%.

### Rodent microglial cells

Primary microglial cells were prepared from the whole brain of 8-week-old male C57BL/6 J mice (CLEA Japan, Inc., Tokyo, Japan) using a magnetic-activated cell sorting as we have reported [13]. Mouse brain tissues were dissociated enzymatically with a neural tissue dissociation kit (Miltenyi Biotec, Auburn, CA) according to the manufacturer’s protocol. Briefly, mouse brain tissues were minced with scalpel, and pre-warmed enzyme mix solution was added to the tissue pieces. After enzymatic dissociation, dissociated tissues were filtered with a 70 μm pore-size cell strainer, and centrifuged. Pellets were re-suspended in MACS buffer (Miltenyi Biotec, Auburn, CA) supplemented with magnetic myelin removal beads (Miltenyi Biotec, Auburn, CA) and incubated for 15 min. Myelin was removed by magnetic separation using LS columns (Miltenyi Biotec, Auburn, CA). To separate primary microglia, cells were magnetically labeled with CD11b MicroBeads (Miltenyi Biotec). CD11b + cells were isolated by LS columns (Miltenyi Biotec), and isolated cells were cultured with DMEM containing 10% FBS, 1% antibiotics, and 1 ng/ml of GM-CSF. The purity of isolated microglia was assessed by immunocytochemical staining for the microglial marker, Iba-1, and > 99% of cells stained positively.

The rat microglial cell line, highly aggressive proliferating immortalized (HAPI) cells [24], was kindly donated by Drs. NP. Morales and F. Hyodo of Kyushu University (Japan). The cells were cultured in DMEM (low glucose; Invitrogen, Waltham, MA), 5% FBS (Hyclone, Logan, UT), 4 mM glutamine (Invitrogen, Waltham, MA), 100,000 U/L Penicillin G, and 100 mg/L streptomycin (Mediatech, Tewksbury, MA), and maintained in 5% CO2 at 37 °C as previously reported [25].

The 6-3 microglial cells were established from neonatal C57BL/6 J (H-2b) mice as described previously [26, 27]. The 6-3 cells were cultured in Eagle’s MEM supplemented with 0.3% NaHCO3, 2 mM glutamine, 0.2% glucose, 10 g/mL insulin, and 10% FCS. Cells were maintained at 37 °C in a 10% CO2, 90% air atmosphere. GM-CSF was supplemented into the culture medium, at a final concentration of 1 ng/mL, to maintain proliferation of the 6-3 cells. Culture media was renewed twice per week.

### Intracellular Ca^2+^ imaging

Intracellular Ca^2+^ imaging using fura-2 AM was performed as reported previously [25–28]. In brief, the experiments were performed in the external standard solution (in mM: 150 NaCl, 5 KCl, 2 CaCl_2_, 1 MgCl_2_, 10 glucose and 10 HEPES, pH 7.4 with Tris-OH) at room temperature (27 °C). For fura-2 excitation the cells were illuminated with two alternating wavelengths, 340 and 380 nm using a computerized system for a rapid dual wavelength Xenon arc. The emitted light was recorded at 510 nm using a cooled CCD camera (Hamamatsu Photonics, Japan). The intracellular Ca^2+^ concentration [Ca^2+^]i was calculated from the ratio (R) of fluorescence recorded at 340 and 380 nm excitation wavelengths for each pixel within a microglial cell boundary. Calibrations (conversion of R340/380 values into calcium concentrations) were performed as described previously [25–28], using a Fura-2 calcium imaging calibration kit (Molecular Probes, Eugene, OR). Basal [Ca^2+^]i was determined from the initial 12 images of each cell recording. A [Ca^2+^]i signal was defined as an increase in R 340/380 with clear time correlation to the application of TNFα. Increase of [Ca^2+^]i in response to TNFα was calculated as the difference between basal [Ca^2+^]i and values obtained at 15 min after a treatment of TNFα. We tested the effect of 100 μM ATP on rodent microglial cells at the end of all experiments and used cells which showed transient intracellular Ca^2+^ elevation for analysis. All data presented were obtained from at least five dishes and three different cell preparations.

### Intracellular NO imaging

The experiments were performed as described previously [25]. The microglial cells were loaded with 10 μM DAF-2DA (4,5-diaminofluorescein diacetate; Sigma-Aldrich, St. Louis, MO), a cell-membrane-permeable dye that binds intracellular NO [29], for 20 min before the measurement. For DAF-2 excitation, the cells were illuminated with a wavelength, 490 nm, using a computerized system. The signal obtained at 490 nm was previously shown to be, among the excitation wavelengths, quantitatively the largest and most representative of change in intracellular NO [30]. The emitted light was collected at 510 nm using a cooled CCD camera. The intracellular DAF-2 fluorescence intensity (F) was recorded for each pixel within a cell boundary. The ratio (F/F0) of fluorescence intensity was estimated from the intensity of fluorescence recorded prior to stimulation (F0).

### Quantitative real time-polymerase chain reaction (qRT-PCR)

qRT-PCR was performed using a LightCycler 480 system (Roche Diagnostics, Mannheim, Germany). The rat HAPI and mouse primary microglial cells were pre-treated with donepezil (5 μM) for 12 h and/or TNFα (3 ng/mL) for 1 h. Cells were washed and the total RNA was extracted using a high pure RNA isolation kit (Roche Diagnostics) according to the manufacturer’s protocol, and was subjected to cDNA synthesis using a transcriptor first strand cDNA synthesis kit (Roche Diagnostics). qRT-PCR was performed with primers (TNFR1: 5’-GTAGCGCTGGAATTGGTTCT-3′, 5’-TGCAAGACATGTCGGAAAGA-3′; TNFR2: 5’-TCCAATTGGTCTGATCGTTG-3′, 5’-AGGTCGCCAGTCCTAACATC-3′; TNFα: 5’-CTGTAGCCCACGTCGTAGC-3′, 3’-TTGAGATCCATGCCGTTG-5′; CD45: 5’-TCAGAAAATGCAACAGTGACAA-3′, 3’-CCAACTGACATCTTTCAGGTATGA-5′; IL-1β: 5’-AGTTGACGGACCCCAAAAG-3′, 3’-AGCTGGATGCTCTCATCAGG-5′; IL-6: 5’-GCTACCAAACTGGATATAATCAGGA-3′, 3’-CCAGGTAGCTATGGTACTCCAGAA-5′; IL-10: 5’-CAGAGCCACATGCTCCTAGA-3′, 3’-TGTCCAGCTGGTCCTTTGTT-5′; TGF-β: 5’-TGGAGCAACATGTGGAACTC-3′, 3’-GTCAGCAGCCGGTTACCA-5′; Arginase: 5’-GAATCTGCATGGGCAACC-3′, 3’-GAATCCTGGTACATCTGGGAAC-5′). Actin-β of Universal Probe Library (Roche Diagnostics) was used as a house-keeping control gene. In Fig. 2, the value of mRNA expression for each sample was automatically calculated as the Ratio by a LightCycler 480 system (Roche Diagnostics, Mannheim, Germany). Ratio = 2^-ΔCp^, ΔCp = Cp(target)-Cp(reference).

### Phagocytosis assay

Phagocytosis was examined via image-base cytometer (TALI, Invitrogen, Waltham, MA) using a phagocytosis assay kit (Cayman Chemical, Ann Arbor, MI) according to the manufacturer’s protocol. Phagocytosis assay kit (IgG FITC) employs latex beads coated with fluorescently-labeled rabbit IgG as a probe for the measurement of the phagocytic process in vitro. Primary microglial cells were cultured in 24-well plates (Corning, Corning, NY) at a density of 4 × 105 cells/ml. We added 50 μl of the latex beads-FITC solution to each well, and incubated the cells in standard culture conditions for 24 h. After harvesting the cells, we measured the fluorescence intensity of FITC using an image-base cytometer.

### Statistics

All statistical analyses were performed with Statistical Package for the Social Sciences (spss) software (version 18.0; IBM, Armonk, NY). All quantified data represent a mean ± SEM. Statistical significance was determined by ANOVA and Tukey’s post hoc test when more than two groups were compared, and Student’s *t* test when one group was compared to the control group. *p* < 0.05 was considered significant.

## Results

### Pretreatment with donepezil suppressed the TNFα-induced sustained intracellular Ca^2+^ elevation in rodent microglial cells

**Fig. 1:**
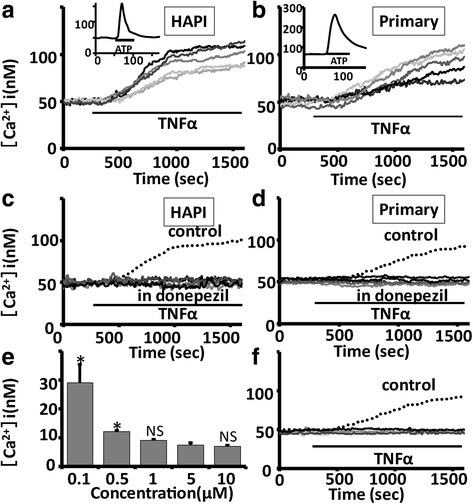
Pretreatment with donepezil suppressed the TNFα-induced sustained intracellular Ca^2+^ elevation in rodent microglial cells. **a**, **b** Five representative traces showing a treatment of 3 ng/mL TNFα-induced sustained increase in [Ca^2+^]i in rat HAPI (**a**) and mouse primary (**b**) microglial cells. (**a**, **b** inset) The inset shows a 100 μM ATP-induced transient increase in [Ca^2+^]i in rat HAPI (**a**) and mouse primary (**b**) microglial cells. The average trace of 15 [Ca^2+^]i traces in response to ATP is shown. **c**, **d** Five representative traces showing that pretreatment with donepezil significantly inhibited the elevation of [Ca^2+^]i induced by TNFα in rat HAPI (**c**) and mouse primary (**d**) microglial cells. Both HAPI (**c**) and primary (**d**) microglial cells were pretreated with 5 μM donepezil for 12 h. **e** Dose-response effect of different concentrations of donepezil on the amplitude of [Ca^2+^]i increase obtained 15 min after TNFα treatment in mouse 6-3 microglial cells. * < 0.05 vs 5 μM donepezil, NS > 0.05 vs 5 μM donepezil. **f** Five representative traces showing that TNFα could not induce sustained intracellular Ca^2+^ elevation in the presence of neutralizing TNFR1 antibody using mouse primary microglial cells. Dotted line is the average trace of control

In the present study, we observed that TNFα (3 ng/mL) induces sustained increase in intracellular Ca^2+^ in rodent microglial cells (Fig. 1a, b) as previously reported [25]. The increase in intracellular Ca^2+^ was sustained for > 50 min even after the washout of TNFα until the end of recording. In contrast, a brief (1 min) application of 100 μM ATP rapidly induced a transient intracellular Ca^2+^ elevation in rodent microglial cells (Fig. 1a, b, inset). We next examined whether pretreatment with donepezil affects the TNFα-induced sustained intracellular Ca^2+^ elevation in rodent microglial cells. The HAPI microglial cells were pretreated with 5 μM donepezil for 12 h. Pretreatment with donepezil significantly inhibited the elevation of [Ca^2+^]i induced by TNFα in rat HAPI microglial cells (38.9 ± 3.3 nM, *n* = 870 in control; 7.8 ± 1.1 nM, *n* = 494 cells; *p* < 0.001; Fig. 1c). In 6-3 murine microglial cells which were pretreated with 5 μM donepezil for 12 h, TNFα (3 ng/mL) also induced a gradual increase in [Ca^2+^]i (data not shown). However, pretreatment of donepezil significantly reduced the amplitude of TNFα-induced increase in [Ca^2+^]i at 15 min after a treatment of TNFα in 6-3 microglial cells (159.6 ± 38 nM, *n* = 37 in control; 69.3 ± 23.0 nM, *n* = 57 in 5 μM donepezil; *p* < 0.05). We also observed that pretreatment with donepezil significantly inhibited the elevation of [Ca^2+^]i induced by TNFα in mouse primary microglial cells (Fig. 1d). These suggest that pretreatment with donepezil suppressed the TNFα-induced increase in [Ca^2+^]i in rodent microglial cells. We have tested the effect of donepezil alone on intracellular Ca^2+^ mobilization in both rat HAPI and mouse primary microglial cells, and found that donepezil alone did not affect intracellular Ca^2+^ mobilization (Additional file 1: Figure S1). In addition, donepezil applied after the onset of TNFα-induced intracellular Ca^2+^ elevation did not affect [Ca^2+^]i in mouse primary microglial cells (Additional file 2: Figure S2).

### Pretreatment with donepezil suppressed the TNFα-induced increase in [Ca^2+^]i in rodent microglial cells through the PI3K pathway

**Fig. 2:**
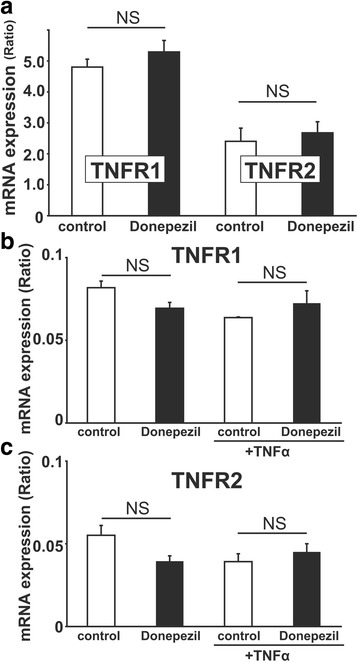
Pretreatment with donepezil did not suppress the mRNA expression of both TNFR1 and TNFR2 in rodent microglial cells. **a**–**c** Bar graphs showing that pretreatment with 5 μM donepezil for 12 h did not affect the mRNA expression of both TNFR1 and TNFR2 in rat HAPI (**a**) and mouse primary (**b**, **c**) microglial cells we used. In addition, pretreatment with donepezil did not suppress the mRNA expression of both TNFR1 (**b**) and TNFR2 (**c**) stimulated by TNFα treatment (3 ng/mL) in mouse primary microglial cells. NS > 0.1 vs control. Note that the unit of TNFR1 is 10 to the minus 3rd power in **a**

TNFα induces intracellular effects through low-affinity 55 kDa type-1 receptors (TNFR1) and high-affinity 75 kDa type-2 receptors (TNFR2) and rodent microglial cells are shown to express both TNFR1 and TNFR2 [31, 32]. We also found the mRNA expression of both TNFR1 and TNFR2 in rat HAPI (Fig. 2a) and mouse primary (Fig. 2b, c) microglial cells using qRT-PCR. In addition, pretreatment with donepezil did not suppress the mRNA expression of both TNFR1 and TNFR2 in rat HAPI (TNFR1, 4.80 ± 0.25, *n* = 4 in control; 5.29 ± 0.37, *n* = 4 in donepezil; *p* = 0.16; TNFR2, 2.41 ± 0.42, *n* = 4 in control; 2.67 ± 0.35, *n* = 4 in donepezil; *p* = 0.32; Fig. 2a) and mouse primary (TNFR1, 0.081 ± 0.004, *n* = 4 in control; 0.069 ± 0.004, *n* = 4 in donepezil; *p* = 0.27; TNFR2, 0.055 ± 0.006, *n* = 4 in control; 0.040 ± 0.003, *n* = 4 in donepezil; *p* = 0.15; Fig. 2b, c) microglial cells we used. We also observed that pretreatment with donepezil did not suppress the mRNA expression of both TNFR1 and TNFR2 stimulated by TNFα treatment (3 ng/mL) in mouse primary microglial cells (TNFR1, 0.064 ± 0.0003, *n* = 4 in control; 0.072 ± 0.007, *n* = 4 in donepezil; *p* = 0.55; Fig. 2b; TNFR2, 0.039 ± 0.005, *n* = 4 in control; 0.045 ± 0.005, *n* = 4 in donepezil; *p* = 0.82; Fig. 2c). These suggest that pretreatment with donepezil suppressed the TNFα-induced increase in [Ca^2+^]i not through the down-regulation of TNFRs mRNA expression in rodent microglial cells.

In addition, we have tested the effect of TNFα in the presence of neutralizing TNFR1 antibody [33], and found that TNFα could not induce sustained intracellular Ca^2+^ elevation in mouse primary microglial cells. These suggest that TNFR1 has a critical role in TNFα-induced intracellular Ca^2+^ elevation (Fig. 1f).

**Fig. 3:**
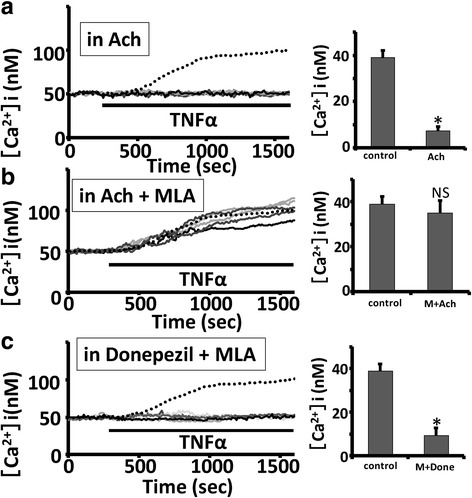
Pretreatment with acetylcholine but not donepezil suppressed the TNFα-induced intracellular Ca^2+^ elevation through the nicotinic α7 receptors in rodent microglial cells. **a**, **b** Five representative traces showing pretreatment with 10 μM acetylcholine (ACh) for 12 h suppressed the TNFα-induced intracellular Ca^2+^ elevation in rat HAPI microglial cells. In contrast, pretreatment with 10 μM ACh and 10 nM MLA, a selective inhibitor of α7nAChRs, did not suppress the TNFα-induced intracellular Ca^2+^ elevation in rat HAPI microglial cells. **c** Five representative traces showing pretreatment with 5 μM donepezil and 10 nM MLA suppressed the TNFα-induced intracellular Ca^2+^ elevation in rat HAPI microglial cells. In each panel, dotted line is the average trace of control. In each panel, bar graphs showing the effect of different manipulations on the amplitude of TNFα-induced increase in [Ca^2+^]i obtained 15 min after TNFα treatment in rat HAPI microglial cells (values are the mean + SEM). * < 0.05 vs control, NS > 0.01 vs control

Microglial cells express α7 nicotinic acetylcholine receptors (α7nAChRs) and treatment with acetylcholine (ACh) is shown to inhibit the activation of microglia through the α7nAChRs [34]. In this study, we examined the effect of ACh on TNFα-induced intracellular Ca^2+^ elevation in rat HAPI microglial cells. We observed that pretreatment with 10 μM ACh for 12 h suppressed the TNFα-induced intracellular Ca^2+^ elevation in rat HAPI microglial cells (7.4 ± 1.7 nM, *n* = 197 cells; *p* < 0.05 vs control; Fig. 3a). In contrast, pretreatment with 10 μM ACh and 10 nM methyllycaconitine (MLA), a selective inhibitor of α7nAChRs [35], did not suppress the TNFα-induced intracellular Ca^2+^ elevation in rat HAPI microglial cells (35.0 ± 5.5 nM, *n* = 195 cells; *p* = 0.16 vs control; Fig. 3b). These suggest that pretreatment with ACh suppressed the TNFα-induced intracellular Ca^2+^ elevation through α7nAChRs in rodent microglial cells.

Pretreatment with donepezil is shown to inhibit the production of NO induced by LPS in BV-2 microglial cells. However, pretreatment with donepezil and MLA also significantly inhibits the production of NO induced by LPS, suggesting that donepezil-induced suppression of microglial activation does not depend on α7nAChRs [21]. We also tested whether pretreatment with donepezil suppressed the TNFα-induced increase in [Ca^2+^]i through α7nAChRs in rodent microglial cells. Pretreatment with 5 μM donepezil and 10 nM MLA for 12 h significantly suppressed the TNFα-induced intracellular Ca^2+^ elevation in rat HAPI microglial cells (9.5 ± 3.3 nM, *n* = 190 cells; *p* < 0.05 vs control; Fig. 3c). Altogether, these suggest that pretreatment with ACh but not donepezil suppressed the TNFα-induced intracellular Ca^2+^ elevation through the α7nAChRs in rodent microglial cells.

Donepezil is also known to be an agonist of sigma-1 receptors with high affinity [36]. Sigma-1 receptors are highly expressed in rodent microglial cells [37]. Using primary microglial cells prepared from rats, pretreatment with 1,3-di-o-tolylguanidine (DTG), an agonist of sigma-1 receptors, is shown to suppress both the release of pro-inflammatory cytokines and increase in [Ca^2+^]i induced by ATP treatment [38]. We next examined whether sigma-1 receptors are involved in the donepezil-induced suppression of the TNFα-mediated intracellular Ca^2+^ elevation in rodent microglial cells. As shown in Additional file 3: Figure S3, pretreatment with 5 μM donepezil and 10 μM BD1047, an antagonist of sigma-1 receptors [39], for 12 h significantly inhibited the elevation of [Ca^2+^]i induced by TNFα in rat HAPI microglial cells (3.4 ± 1.7 nM, *n* = 300; *p* < 0.01 vs control; Additional file 3: Figure S3). Pretreatment of BD1047 alone did not suppress the TNFα-induced increase in [Ca^2+^]i (data not shown). We also found that pretreatment with 5 μM donepezil and 10 μM BD1063, another antagonist of sigma-1 receptors [39], for 12 h significantly inhibited the elevation of [Ca^2+^]i induced by TNFα in rat HAPI microglial cells (data not shown). These suggest that sigma-1 receptors were not important for the donepezil-induced suppression of the TNFα-mediated intracellular Ca^2+^ elevation in rodent microglial cells.

**Fig. 4:**
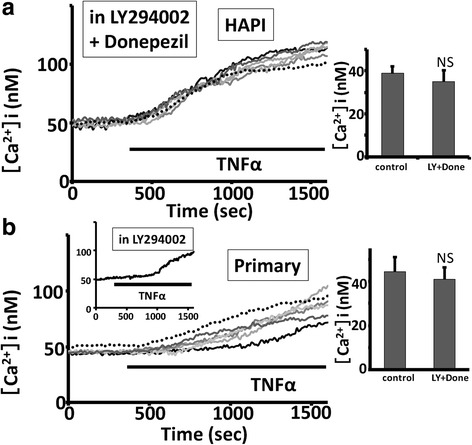
Pretreatment with donepezil suppressed the TNFα-induced increase in [Ca^2+^]i in rodent microglial cells through the PI3K pathway. **a**, **b** Five representative traces showing pretreatment with 5 μM donepezil and 10 μM LY294002, a selective PI3K inhibitor, for 12 h did not inhibited the elevation of [Ca^2+^]i induced by TNFα in rat HAPI (**a**) and mouse primary (**b**) microglial cells. The inset in Fig. 5b shows pretreatment with LY294002 alone did not affect the TNFα-induced increase in [Ca^2+^]i in mouse primary microglial cells. The average trace determined from 5 representative traces of [Ca^2+^]i in response to TNFα was shown. In each panel, dotted line is the average trace of control. In each panel, bar graphs showing the amplitude of TNFα-induced increase in [Ca^2+^]i obtained 15 min after TNFα treatment in rat HAPI (**a**) and mouse primary (**b**) microglial cells (values are the mean + SEM). NS > 0.1 vs control

In the CNS, donepezil protects neurons against neurotoxicity induced by glutamates through the phosphatidylinositol 3-kinase (PI3K)/Akt pathway [40]. In addition, PI3K/Akt pathway is important for the suppression of production of pro-inflammatory cytokines induced by LPS in rodent microglial cells [41]. We next examined whether PI3K is involved in the donepezil-induced suppression of the TNFα-mediated intracellular Ca^2+^ elevation in rodent microglial cells. As shown in Fig. 4a, b, pretreatment with 5 μM donepezil and 10 μM LY294002, a selective PI3K inhibitor [42], for 12 h did not inhibit the elevation of [Ca^2+^]i induced by TNFα in both rat HAPI (34.9 ± 5.5 nM, *n* = 300; *p* = 0.37 vs control; Fig. 4a) and mouse primary (Fig. 4b) microglial cells.

**Fig. 5:**
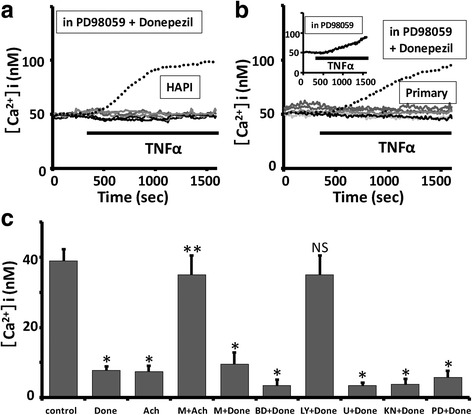
PLC, CaMK2, and MAPK/ERK were possibly not involved in the donepezil-induced suppression of the TNFα-mediated intracellular Ca^2+^ elevation in rodent microglial cells. **a**, **b** Five representative traces showing pretreatment with 5 μM donepezil and 10 μM PD98059, a MAP kinase/extracellular signal-regulated kinase (ERK) inhibitor, for 12 h significantly suppressed the TNFα-induced intracellular Ca^2+^ elevation in rat HAPI (**a**) and mouse primary (**b**) microglial cells. The inset in Fig. 5b shows pretreatment with PD98059 alone did not affect the TNFα-induced increase in [Ca^2+^]i in mouse primary microglial cells. The average trace determined from 5 representative traces of [Ca^2+^]i in response to TNFα was shown. In each panel, dotted line is the average trace of control. **c** Histogram summarizing the effect of different manipulations on the amplitude of TNFα-induced increase in [Ca^2+^]i obtained 15 min after TNFα treatment in rat HAPI microglial cells (values are the mean + SEM). * < 0.05 vs control, NS > 0.01 vs control, ** < 0.05 vs Ach

In rodent microglial cells, ADP, a selective agonist for P2Y12 receptors, is shown to promote chemotaxis through the phospholipase C (PLC)-mediated elevation of [Ca^2+^]i followed by the activation of PI3K pathway [43]. We observed that pretreatment with 5 μM donepezil and 1 μM U73122, a membrane-permeable specific PLC inhibitor [44], for 12 h significantly suppressed the TNFα-induced intracellular Ca^2+^ elevation in rat HAPI microglial cells (3.4 ± 0.8 nM, *n* = 186 cells; *p* < 0.01 vs control; Fig. 5c).

Although CaMKII has also important roles in the regulation of microglial functions [45], we found that pretreatment with 5 μM donepezil and 1 μM KN-62, a CAMKII inhibitor [46], for 12 h significantly suppressed the TNFα-induced intracellular Ca^2+^ elevation in rat HAPI microglial cells (3.8 ± 1.5 nM, *n* = 160 cells; *p* < 0.01 vs control; Fig. 5c).

Treatment with donepezil is shown to increase the expression of phosphorylated extracellular signal-regulated kinase1/2 (p-ERK1/2) in the hippocampus isolated from aged mice [47]. In addition, the activation of MAPK/ERK is important for the production of NO induced by LPS [48] or by IFNγ [28] in rodent microglial cells. Sodium butyrate, an anti-inflammatory agent, downregulates the expression of iNOS induced by IFNγ through the inhibition of ERK in rodent microglial cells [49]. Thus, we tested the possible involvement of MAPK/ERK in the donepezil-induced suppression of the TNFα-mediated intracellular Ca^2+^ elevation in rodent microglial cells. As shown in Fig. 5a, b, pretreatment with 5 μM donepezil and 10 μM PD98059, a MAP kinase/extracellular signal-regulated kinase (ERK) inhibitor [50], for 12 h significantly suppressed the TNFα-induced intracellular Ca^2+^ elevation in both rat HAPI (5.7 ± 1.9 nM, *n* = 180 cells; *p* < 0.01 vs control; Fig. 5a, c) and mouse primary (Fig. 5b) microglial cells.

Altogether, these suggest that pretreatment with donepezil suppressed the TNFα-induced increase in [Ca^2+^]i in rodent microglial cells through the PI3K pathway. In addition, PLC, MAPK/ERK, and CaMKII were possibly not involved in the donepezil-induced suppression of the TNFα-mediated intracellular Ca^2+^ elevation in rodent microglial cells.

Donepezil is shown to protect against neurotoxicity induced by glutamate through the PI3K/Akt pathway. McMullan et al. reported that group-II and –III mGluR activation attenuates a potentially neurotoxic export of glutamate from activated microglia [51]. We observed that pretreatment with 5 μM donepezil and 30 μM MSPG, a group-II and-III antagonist, for 12 h significantly suppressed the TNFα-induced intracellular Ca^2+^ elevation in mouse primary microglial cells (Additional file 4: Figure S4). These suggest that group-II and –III mGluRs were possibly not involved in the donepezil-induced suppression of the TNFα-mediated intracellular Ca^2+^ elevation in rodent microglial cells.

### Pretreatment with donepezil suppressed the production of NO induced by TNFα in rodent microglial cells through the PI3K pathway

**Fig. 6:**
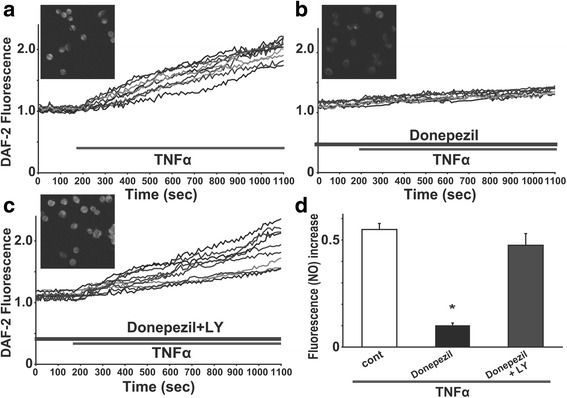
Pretreatment with donepezil suppressed the production of NO induced by TNFα in rodent microglial cells through the activation of PI3K pathway. **a** Ten representative traces showing the treatment of 0.1 ng/mL TNFα induced the increase in the DAF-2 fluorescence in rodent microglial cells. **b** Ten representative traces showing 12 h pretreatment with 5 μM donepezil suppressed the TNFα-induced increase in the DAF-2 fluorescence in rodent microglial cells. **c** Ten representative traces showing 12 h pretreatment with both 5 μM donepezil and 10 μM LY294002 did not suppress the TNFα-induced increase in the DAF-2 fluorescence in rodent microglial cells. Representative images (15 min after a treatment of TNFα) are shown in insets of **a**, **b,** and **c**. **d** Bar graphs showing that pretreatment with donepezil suppressed the production of NO induced by TNFα treatment and the PI3K pathway could be important for the donepezil-induced suppression of the NO production in rodent microglial cells

We next tested the effect of TNFα on intracellular NO mobilization, using DAF-2 imaging to detect endogenously produced NO in rodent microglial cells. An application of 0.1 ng/mL TNFα induced a gradual increase in DAF-2 fluorescence in rodent microglial cells (*n* = 189; Fig. 6a) as previously reported [25]. The reaction between DAF-2 and NO is shown to be irreversible and the accumulated level of DAF-2 fluorescence reflects the total amount of intracellular NO production [29, 52]. We observed that the increase in intracellular DAF-2 fluorescence was sustained for > 50 min even after the washout of TNFα until the end of recording. Additionally, in the presence of 50 μM L-N6-(1-iminoethyl)lysine (L-NIL), a membrane-permeable selective inhibitor of inducible nitric oxide synthase (iNOS; [53]), TNFα failed to elevate the DAF-2 fluorescence in rodent microglial cells (*n* = 60; data not shown).

We measured the effect of 12 h pretreatment with donepezil (5 μM) on the production of intracellular NO induced by TNFα in rodent microglial cells. In rodent microglial cells which were pretreated with donepezil for 12 h, TNFα (0.1 ng/mL) also induced a gradual increase in the DAF-2 fluorescence (Fig. 6b). However, pretreatment of donepezil significantly reduced the amplitude of TNFα-induced increase in the DAF-2 fluorescence at 15 min after a treatment of TNFα in rodent microglial cells (0.549 ± 0.028, *n* = 189 in control; 0.101 ± 0.011, *n* = 70 in 5 μM donepezil; *p* < 0.001; Fig. 6d). In contrast, 12 h pretreatment of both donepezil (5 μM) and LY294002 (10 μM) did not reduce the amplitude of TNFα-induced increase in the DAF-2 fluorescence in rodent microglial cells (0.549 ± 0.028, *n* = 189 in control; 0.475 ± 0.054, *n* = 45 in donepezil and LY294002; *p* = 0.12; Fig. 6c, d). These suggest that pretreatment with donepezil suppressed the production of NO induced by TNFα. In addition, activation of PI3K pathway could be important for the donepezil-induced suppression of NO production in rodent microglial cells.

**Fig. 7:**
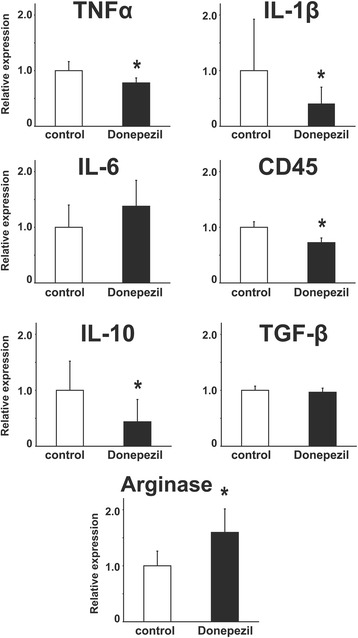
Bar graphs showing the effects of pretreatment with donepezil on mRNA expression of TNFα, IL-1β, IL-6, CD45, IL-10, TGF-β, and Arginase in mouse primary microglial cells. * < 0.05 vs control

Moreover, we have tested the effects of pretreatment of 5 μM donepezil for 12 h on some pro-inflammatory and anti-inflammatory phenotypes in mouse primary microglial cells using qRT-PCR. Although donepezil significantly suppressed the mRNA expression of TNFα, IL-1β and CD45, representative markers of pro-inflammatory phenotypes, we did not observe donepezil augment the mRNA expression of anti-inflammatory phenotypes in mouse primary microglial cells we used (Fig. 7).

### Pretreatment with donepezil promoted phagocytic activity of mouse primary microglial cells through the PI3K pathway

**Fig. 8:**
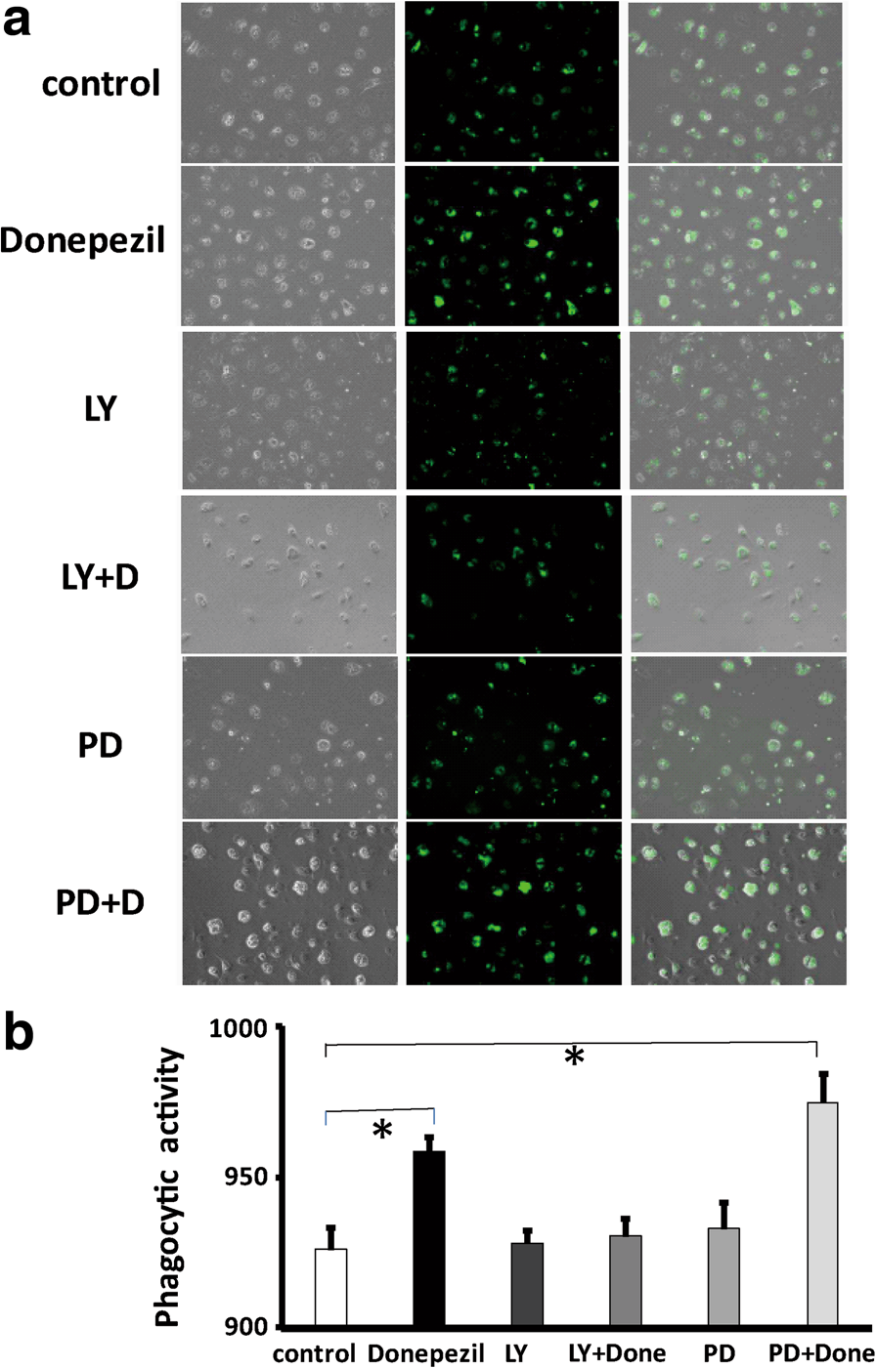
Pretreatment with donepezil promoted phagocytic activity of mouse primary microglial cells through the PI3K but not MAPK/ERK pathway. **a** Representative images showing that pretreatment with donepezil promoted phagocytic activity of mouse primary microglial cells through the PI3K but not MAPK/ERK pathway. Images shows bright field (left), FITC (middle) and Overlay (right), respectively. **b** Bar graphs showing that pretreatment of 5 μM donepezil for 12 h enhanced the phagocytic activity of mouse primary microglial cells to about 105% control. In addition, pretreatment with donepezil and LY294002 (10 μM) but not PD98059 (10 μM) for 12 h significantly suppressed the donepezil-induced promotion of phagocytic activity

Finally, we also investigated whether pretreatment of donepezil affects the phagocytic activity of rodent microglial cells. The phagocytosis assay showed that pretreatment of 5 μM donepezil for 12 h enhanced the beads phagocytic activity of mouse primary microglial cells (*n* = 3; Fig. 8a, b). In addition, pretreatment with donepezil and LY294002 (10 μM) but not PD98059 (10 μM) for 12 h significantly suppressed the donepezil-induced promotion of phagocytic activity (*n* = 3; Fig. 8a, b). These suggest that the PI3K but not MAPK/ERK pathway could be important for the donepezil-induced promotion of phagocytic activity in mouse primary microglial cells.

## Discussion

In the present study, we observed that pretreatment with donepezil suppressed the TNFα-induced sustained intracellular Ca^2+^ elevation in rodent microglial cells. On the other hand, pretreatment with donepezil did not suppress the mRNA expression of both TNFR1 and TNFR2 in rodent microglia we used. Pretreatment with acetylcholine but not donepezil suppressed the TNFα-induced intracellular Ca^2+^ elevation through the nicotinic α7 receptors (α7nAChRs) in rodent microglial cells. In addition, sigma 1 receptors were not involved in the donepezil-induced suppression of the TNFα-mediated intracellular Ca^2+^ elevation. Pretreatment with donepezil suppressed the TNFα-induced increase in [Ca^2+^]i through the PI3K pathway in rodent microglial cells. In contrast, PLC, MAPK/ERK and CaMKII were possibly not involved in the donepezil-induced suppression of the TNFα-mediated intracellular Ca^2+^ elevation. Using DAF-2 imaging, we also found that pretreatment with donepezil suppressed the production of NO induced by TNFα treatment and the PI3K pathway could be important for the donepezil-induced suppression of NO production in rodent microglial cells. Finally, the phagocytosis assay showed that pretreatment with donepezil promoted phagocytic activity of mouse primary microglial cells through the PI3K but not MAPK/ERK pathway. To the best of our knowledge, this is the first report showing that pretreatment with donepezil could directly modulate intracellular Ca^2+^ mobilization and phagocytic activity in rodent microglial cells.

The major mechanism of donepezil’s effects is to inhibit AChE activity thereby increasing the ACh levels. ACh then promotes neuroprotective effects against glutamate-induced excitotoxicity through the α7nAChRs [54] or the PI3K/Akt signaling pathway [19]. Microglial cells also express α7nAChRs and treatment with ACh is shown to inhibit the activation of microglia through the α7nAChRs [34]. Specifically, De Simone et al. found that treatment of ACh or nicotine dose-dependently suppressed the release of TNFα induced by LPS in rodent microglial cells, showing the existence of cholinergic anti-inflammatory pathway mediated by α7nAChRs in the brain. In the present study, we also observed that pretreatment with ACh suppressed the TNFα-induced intracellular Ca^2+^ elevation in rodent microglial cells. In addition, pretreatment with ACh and MLA, antagonist of α7nAChRs, did not suppress the TNFα-induced intracellular Ca^2+^ elevation, suggesting that ACh suppressed the TNFα-induced intracellular Ca^2+^ elevation through α7nAChRs in rodent microglial cells we used. These suggest that AChE inhibitors including donepezil exert their therapeutic effects through both potentiation of ACh-mediated neuronal transmission and anti-inflammatory actions in the brain of AD [55]. Unexpectedly, Hwang et al. found that treatment with MLA did not affect the inhibitory effects of donepezil on production of both TNFα and NO induced by LPS in rodent microglial cells [21]. We also observed that pretreatment with donepezil and MLA significantly suppressed the TNFα-induced intracellular Ca^2+^ elevation in rodent microglial cells (Fig. 3c). These suggest that pretreatment with ACh but not donepezil suppressed the TNFα-induced intracellular Ca^2+^ elevation through the α7nAChRs in rodent microglial cells. Recently, Arikawa et al. also showed that anti-inflammatory effects of donepezil on murine macrophage cells are not dependent on its AChE inhibition [56]. Thus, donepezil appears to modulate the microglial functions independently of α7nACh receptors mediated signaling.

Some reports have previously shown that the neuroprotective effect of donepezil against glutamate neurotoxicity is mediated through the activation of PI3K/Akt pathway [19, 40, 57]. Although the activation of PI3K/Akt pathway is important for the suppression of production of pro-inflammatory cytokines induced by LPS in rodent microglial cells [41], whether the effects of donepezil on microglial function also depend on the activation of PI3K/Akt pathway remains elusive. We observed that activation of PI3K/Akt pathway was important for both effects of donepezil on mobilization of intracellular Ca^2+^ and production of NO in rodent microglial cells.

Galantamine, another AChE inhibitor, stimulates the microglial phagocytosis of Aβ peptides through the activation of CaMKII pathway [58]. In contrast, the effect of donepezil on phagocytic activity of microglial cells remains to be established. In the present study, the phagocytosis assay showed that pretreatment of donepezil for 12 h enhanced the phagocytic activity of mouse primary microglial cells. The PI3K but not MAPK/ERK pathway could be important for the donepezil-induced promotion of phagocytic activity in mouse primary microglial cells. Interestingly, both PI3K and MAPK/ERK pathways are not involved in the effect of galantamine on microglial phagocytosis of Aβ peptides [58]. Thus, although AChE inhibitors promote the phagocytic activity of microglial cells, underlying intracellular pathways might differ among AChE inhibitors. In addition, using organotypic hippocampal slice cultures, Katayama et al. recently reported that p38 MAP kinase but not MAPK/ERK is important for microglial phagocytosis of injured neurons induced by NMDA-receptors mediated excitotoxicity [59]. We need to further examine the mechanism underlying the donepezil-induced promotion of phagocytic activity in rodent microglial cells. In particular, the mediating receptor by which donepezil affects intracellular PI3K but not MAPK/ERK pathway remains to be elucidated.

Elevation of intracellular Ca^2+^ is important for the activation of microglia, including proliferation, migration, ramification, de-ramification and release of NO, pro-inflammatory cytokines and neurotrophic factors [1, 5]. We have recently reported that pretreatment with BDNF suppressed the production of NO induced by TNFα possibly through the modulation of intracellular Ca^2+^ mobilization in rodent microglial cells [25, 26]. As one of underlying mechanisms of aging and AD, dysregulation of intracellular Ca^2+^ homeostasis has also been proposed as a common cause of neural dysfunction [15, 16]. However, some reports suggest that dysregulation of intracellular Ca^2+^ is not restricted to neurons but represents a global phenomenon affecting glia including microglia cells in the brain of AD patients [17]. TNFα has pro-inflammatory effects by induction of nuclear factor kappa B (NF-κB) [60]. In cultured astrocytes prepared from mice, elevation of intracellular Ca^2+^ is important for the TNFα-induced PARP-1 phosphorylation resulting in the activation of NF-κB [33]. In the present study, we observed that pretreatment with donepezil suppressed the TNFα-induced sustained intracellular Ca^2+^ elevation in rodent microglial cells. These suggest that donepezil could modulate the transcriptional activation of NF-κB induced by TNFα through the suppression of intracellular Ca^2+^ mobilization in rodent microglial cells.

In the brain of patients with AD, the expression of iNOS is found to be elevated [61] and genetic knockout of iNOS has neuroprotective effects in the mouse model of AD [62]. In addition, Aβ peptides are target of NO because nitration of Aβ peptide at tyrosine 10 is shown to be increase the propensity of Aβ to aggregate in the brain [63]. In the present study, we observed that pretreatment with donepezil suppressed the production of NO induced by TNFα in rodent microglial cells. Thus, in addition to the anti-inflammatory effects, donepezil could prevent the aggregation of Aβ peptides through the suppression of production of NO induced by TNFα in the brain.

One of the major limitations of our observation is the reliance on in vitro work of microglia activation. However, our in vitro work is compatible with the in vivo study showing that donepezil improves cognitive deficits in association with its inhibition of microglial activation using transgenic models of AD [20, 22].

## Conclusions

AD, the most prevalent cause of dementia, is still defined by the combined presence of amyloid and tau, but researchers are gradually moving away from the simple assumption of linear causality as proposed in the original amyloid hypothesis [12]. As the disease progresses, microglial cells aggregate around the Aβ deposits, both in the brains of patients with AD at post-mortem and in transgenic mice models of AD. Their inability to remove amyloid plaques in late disease has been implicated as a cause of neurodegeneration [64]. In addition, free radical such as NO induces oxidative injury and degeneration of microglia during aging. This called microglial senescence is supposed to underlie the pathogenesis of AD, based on the idea that degeneration rather than over-activation of microglia could really contribute to the loss of normal functions of microglia during aging and in the brain of AD [65]. We herein reported that donepezil could directly modulate intracellular Ca^2+^ mobilization and phagocytic activity in rodent microglial cells, which might be important to understand the effect of donepezil in the brain.

## Additional files

Additional file 1: Figure S1. Five representative traces showing that donepezil alone did not affect [Ca^2+^]i in mouse primary microglial cells. (TIFF 901 kb)

Additional file 2: Figure S2. Five representative traces showing that donepezil applied after the onset of TNFα-induced intracellular Ca^2+^ elevation did not affect [Ca^2+^]i in mouse primary microglial cells (TIFF 923 kb)

Additional file 3: Figure S3. Sigma 1 receptors were not involved in the donepezil-induced suppression of the TNFα-mediated intracellular Ca^2+^ elevation in rodent microglial cells. Pretreatment with 5 μM donepezil and 10 μM BD1047, an antagonist of sigma-1 receptors, for 12 h significantly inhibited the elevation of [Ca^2+^]i induced by TNFα in rat HAPI microglial cells. In this panel, the average trace determined from 5 representative traces of [Ca^2+^]i in each condition. Dotted line is the average trace of control. (TIFF 222 kb)

Additional file 4: Figure S4. Five representative traces showing that pretreatment with 5 μM donepezil and 30 μM MSPG, a group-II and-III antagonist, for 12 h significantly suppressed the TNFα-induced intracellular Ca^2+^ elevation in mouse primary microglial cells. (TIFF 1305 kb)

## Abbreviations

AChE: Acetylcholinesterase
AD: Alzheimer’s disease
DAF-2DA: 4,5-diaminofluorescein diacetate
NO: Nitric oxide
PI3K: Phosphatidylinositol-3 kinase
α7nAChRs: α7 nicotinic acetylcholine receptors
