# Supplementary figures and images for: Donepezil suppresses intracellular Ca2+ mobilization through the PI3K pathway in rodent microglia
Source: J Neuroinflammation. 2017 Dec 22;14:258. doi: 10.1186/s12974-017-1033-0 (PMC5741946; doi:10.1186/s12974-017-1033-0)

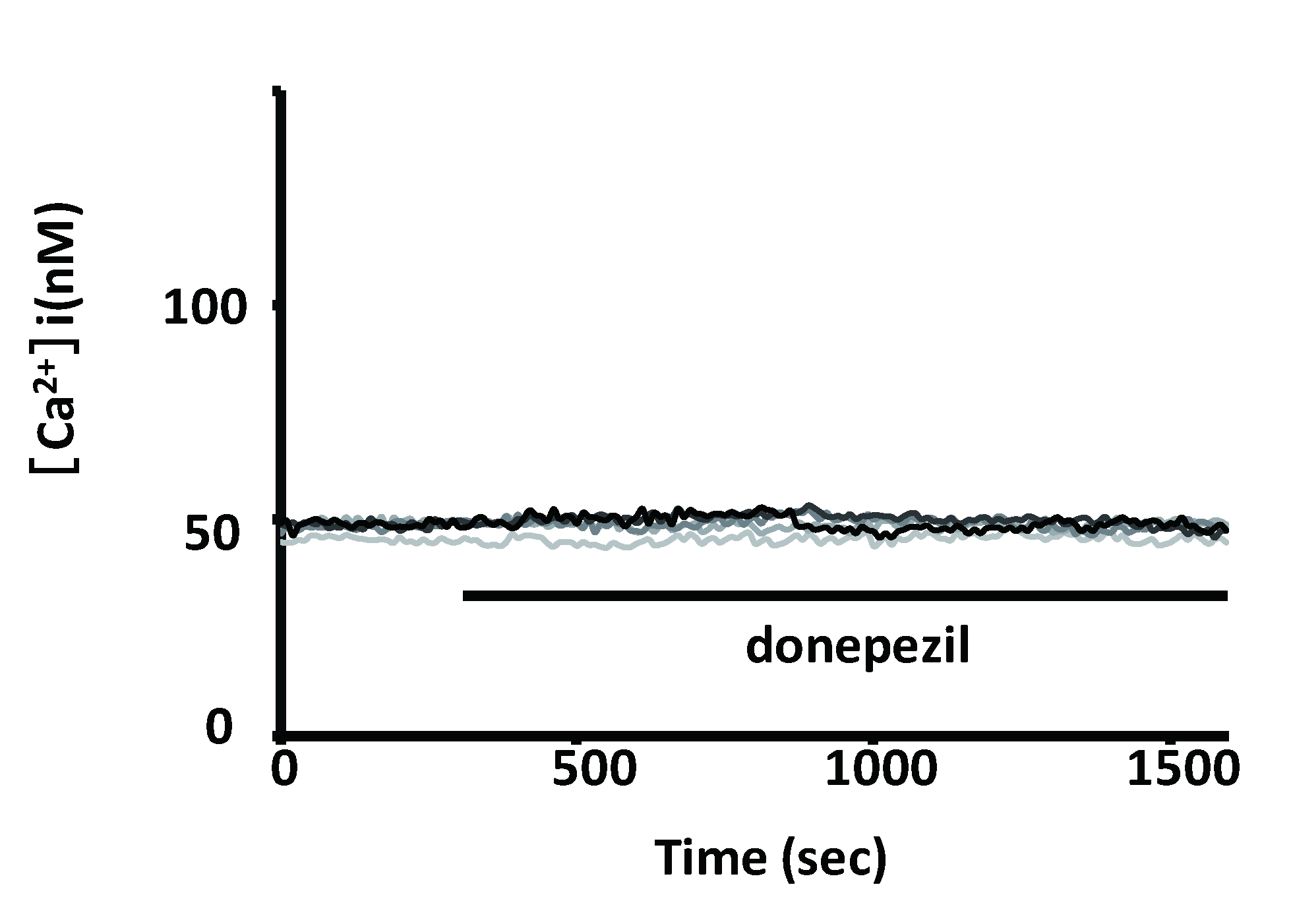

Supplement: Supplementary file 1 — Five representative traces showing that donepezil alone did not affect [Ca2+]i in mouse primary microglial cells. (TIFF 901 kb) [file 12974_2017_1033_MOESM1_ESM.tif]

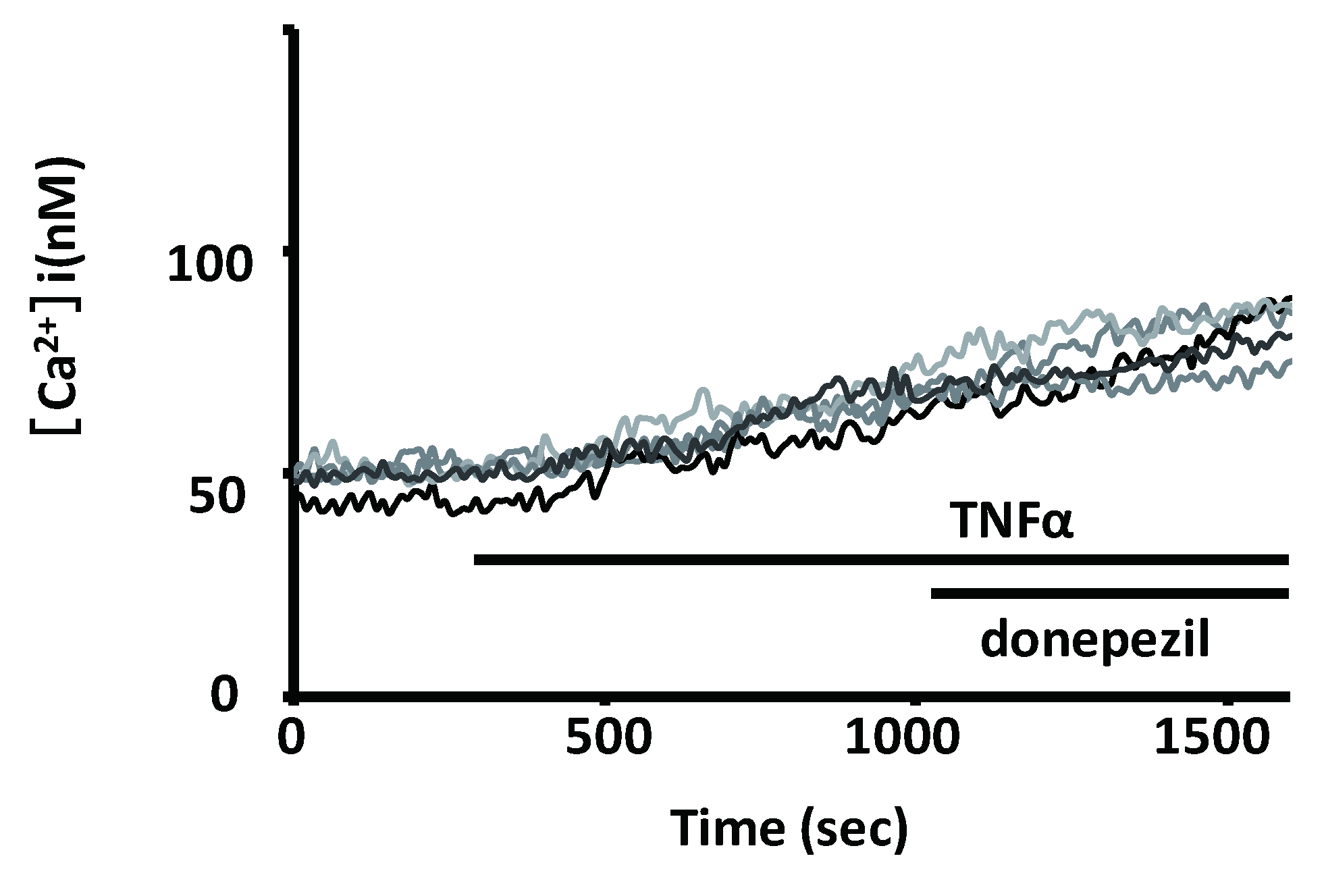

Supplement: Supplementary file 2 — Five representative traces showing that donepezil applied after the onset of TNFα-induced intracellular Ca2+ elevation did not affect [Ca2+]i in mouse primary microglial cells (TIFF 923 kb) [file 12974_2017_1033_MOESM2_ESM.tif]

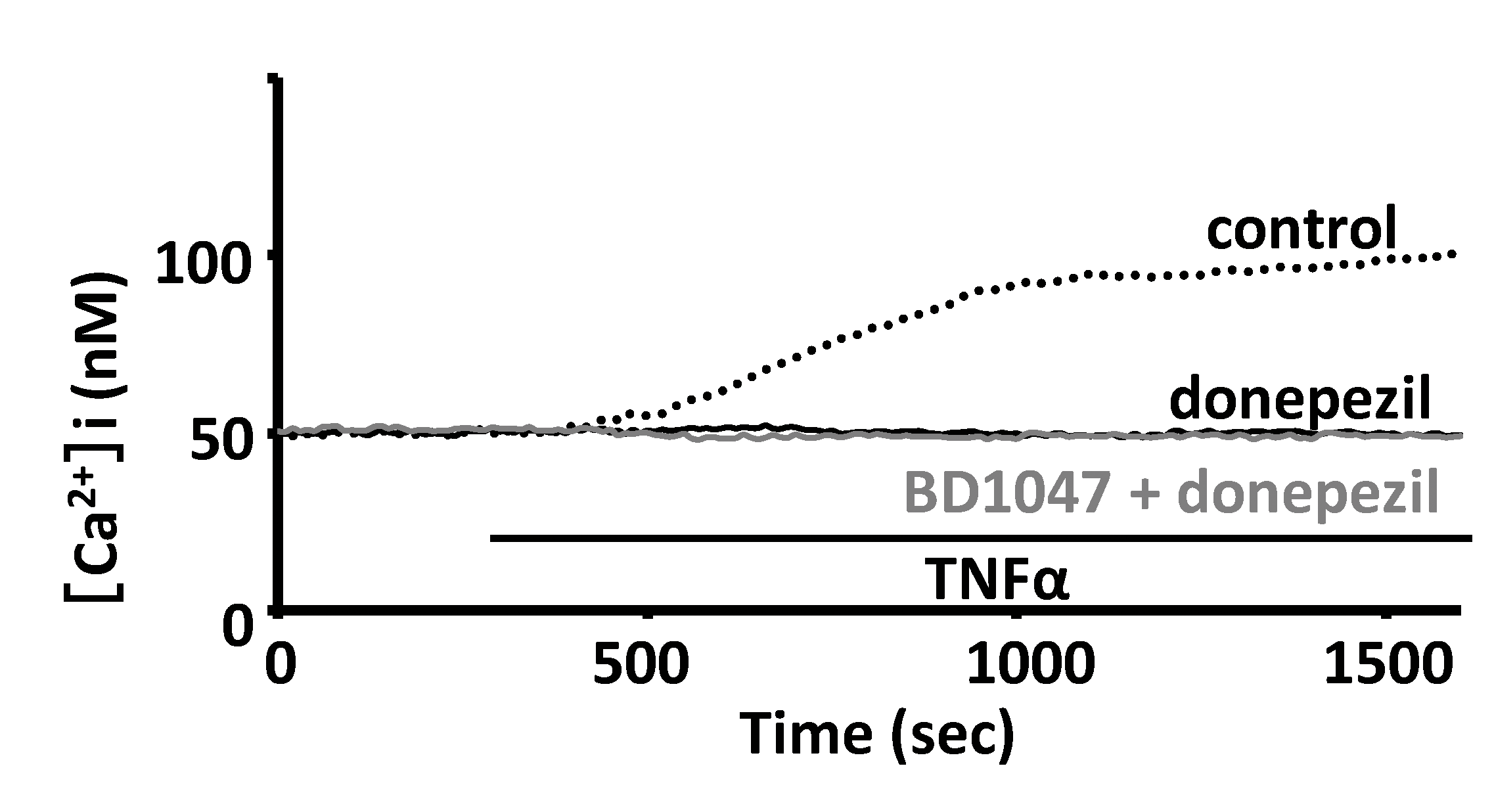

Supplement: Supplementary file 3 — Sigma 1 receptors were not involved in the donepezil-induced suppression of the TNFα-mediated intracellular Ca2+ elevation in rodent microglial cells. Pretreatment with 5 μM donepezil and 10 μM BD1047, an antagonist of sigma-1 receptors, for 12 h significantly inhibited the elevation of [Ca2+]i induced by TNFα in rat HAPI microglial cells. In this panel, the average trace determined from 5 representative traces of [Ca2+]i in each condition. Dotted line is the average trace of control. (TIFF 222 kb) [file 12974_2017_1033_MOESM3_ESM.tif]

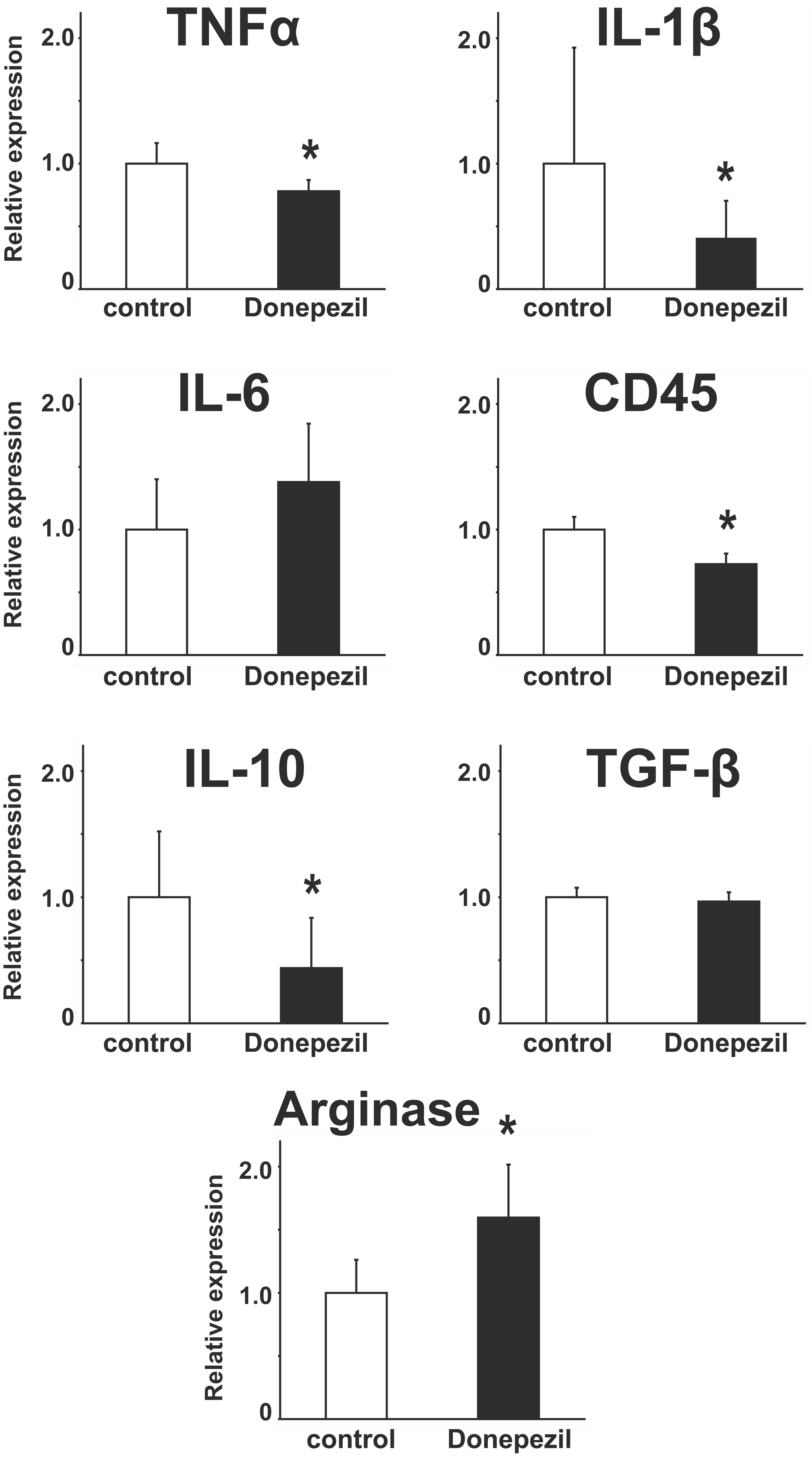

Supplement: Supplementary file 4 — Five representative traces showing that pretreatment with 5 μM donepezil and 30 μM MSPG, a group-II and-III antagonist, for 12 h significantly suppressed the TNFα-induced intracellular Ca2+ elevation in mouse primary microglial cells. (TIFF 1305 kb) [file 12974_2017_1033_MOESM4_ESM.tif]
